# Hydrocortisone rapidly and significantly reduces the IL-6 level in blood and lungs of patients with COVID-19-related ARDS

**DOI:** 10.1186/s13054-024-04887-2

**Published:** 2024-03-28

**Authors:** Antoine Guillon, Youenn Jouan, Arthur Kassa-Sombo, Christophe Paget, Pierre-François Dequin

**Affiliations:** 1 Intensive Care Unit, Tours University Hospital, 2 Bd Tonnellé, 37044 Tours Cedex 9, France; 2 Research Center for Respiratory Diseases, INSERM U1100, University of Tours, Tours, France; 3 Cardiac Surgery Department, Tours University Hospital, Tours, France

Dear Editor,

Increased levels of inflammatory mediators, particularly IL-6, have been observed in conditions like sepsis, acute respiratory distress syndrome (ARDS), and more recently, COVID-19, leading to the exploration of anti-inflammatory treatments. To date, clinical practice guidelines include strong recommendations in favor of a limited number of immunomodulatory for severe COVID-19: corticosteroids, interleukin 6 receptor blockers, and baricitinib [1]. All three may be combined. However, the exact effect of corticosteroids on the main inflammatory cytokines is unknown in critically ill patients with COVID-19. In particular, the pathophysiology of the respiratory failure in COVID-19 is attributed to local immune dysregulation with a lung compartmentalization of inflammatory mediators [2]. The efficacy of corticosteroids to rapidly control the lung inflammation is unknown. The objective of this study was to describe the concentrations in blood and respiratory fluid of IL-6, TNF-α, and IL-1β after a single administration of hydrocortisone, compared to placebo.

We took advantage of two studies performed simultaneously in the same center (CAPE COVID trial and ImPACT study) and performed ancillary analysis of patients included in both studies. The CAPE COVID trial is a randomized, double-blinded, placebo-controlled study assessing the effectiveness of hydrocortisone in patients with COVID-19-related ARDS [3]. The ImPACT study described immunophenotyping in blood and in the supernatants of endotracheal aspirates of severe COVID-19 patients [4]. Briefly, patients aged at least 18 years admitted in ICU and mechanically ventilated for ARDS could be included if they had a biologically confirmed (reverse transcriptase–polymerase chain reaction) COVID-19. The experimental treatment (hydrocortisone or placebo) had to be administered within 24 h of the onset of the first severity criterion (need for mechanical ventilation and/or Pao2/FiO2 < 300 mmHg and/or Pulmonary Severity Index greater than 130) or within 48 h for patients referred from another hospital. Patients receiving vasopressors to correct hypotension related to sedative drugs and mechanical ventilation at high PEEP levels could be included. Principal exclusion criteria were septic shock and do-not-intubate orders. Randomization was centralized and performed electronically. Patients received a continuous intravenous infusion of hydrocortisone at an initial dose of 200 mg/d or its placebo (saline). For the ImPACT study, inflammatory mediators were measured in sera and supernatants of endotracheal aspirates using the Bio-Plex Pro Human cytokines screening panel (Bio-Rad) in a multiplex fluorescent bead assay (Luminex), according to the manufacturer’s instructions. Endotracheal aspirates were collected, then weighed, and incubated in PBS (5 ml/g) with 1 mM dithiothreitol for 30 min at 4 °C under gentle agitation. We thus analyzed samples (from the ImPACT study) that were taken either before or after the start of hydrocortisone or placebo administration (from the CAPE COVID trial).

Twenty patients with COVID-19-related ARDS were enrolled in both studies simultaneously. Patients’ characteristics were (median [quartile 1; quartile 3]), age 64 (57;67) y.o, male/female 2/1, MBI 31 (28;32) kg/m^2^, SAPS2 32 (22;36), and SOFA 4 (2;6). To examine the temporal impact of hydrocortisone or placebo on cytokines concentrations, a linear regression with logarithmic transformation was used. After modeling, trend graphs were generated, along with confidence intervals (Fig. 1). Analysis of these results revealed a statistically significant negative regression coefficient in the hydrocortisone group for IL-6 concentrations. Linear regression was then explored by incorporating an interaction between time and groups. For this purpose, a binary variable was introduced to differentiate between samples taken at negative (before dosing) and positive (after dosing) times. Fitting this regression revealed significant disparities in the temporal influence on hydrocortisone on IL-6 levels, both in blood and in endotracheal aspirate. More specifically, significant tests (*p* < 0.05) associated with the coefficients confirmed the impact of hydrocortisone on IL-6 measurements; the use of analysis of variance corroborated the significance of the results. Consequently, the IL-6 concentration was barely detectable in the blood after the hydrocortisone administration compared to the placebo group (0.45 [0.45 – 2.2] and 98 [40.5 – 284.6] pg/mL, respectively) and was lower in the endotracheal aspirate (2395 [623 – 2946] and 7854 [5273 – 24,713] pg/mL, respectively). No significant temporal variations were observed for TNF-α and IL1-β after hydrocortisone administration.

**Fig. 1:**
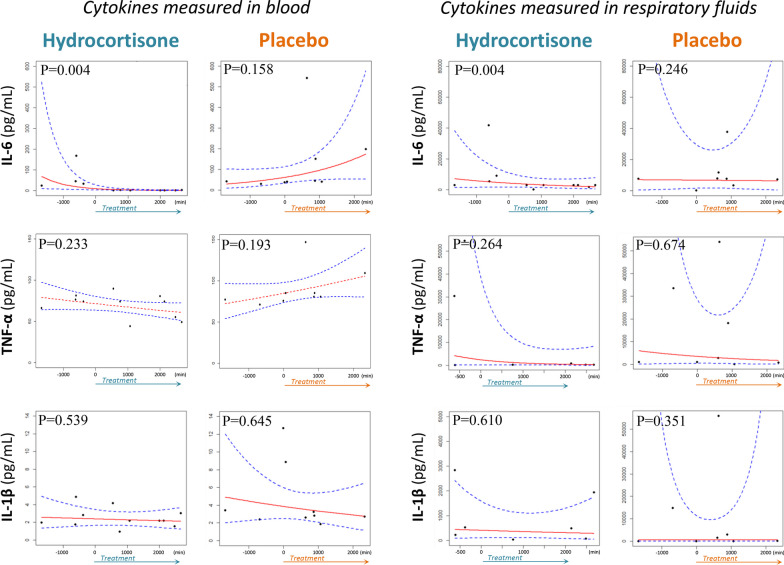
Effect of hydrocortisone treatment on pro-inflammatory cytokine kinetics. IL-6, TNF-α, and IL-1β concentrations were measured in blood and supernatants of endotracheal aspirates of patients with COVID-19-related ARDS before and after the start of hydrocortisone or placebo administration. Red lines represent trend graphs; blue dot lines represent confidence intervals. The *p*-value linked to the regression analysis with interaction evaluates the statistical significance of the observed changes in cytokine concentrations after administering hydrocortisone or a placebo. A *p*-value less than 0.05 indicates a significant effect

Although the relation between steroids and IL-6 is well known, our study provides new insights. (i) It is the first and only demonstration showing the effectiveness of steroids in reducing IL-6 levels in COVID-19-related ARDS with a randomized, double-blinded, placebo-controlled design. (ii) The IL-6 concentration becomes negligible in the blood after the start of hydrocortisone treatment. (iii) There is a reduction of more than two times the initial IL-6 level in the lungs immediately after the intravenous infusion of hydrocortisone. In summary, hydrocortisone rapidly and significantly reduces the IL-6 level in blood and lungs of patients with COVID-19-related ARDS patients.

## Data Availability

The datasets generated and/or analyzed during the current study are not publicly available but are available from the corresponding author on reasonable request.

## Abbreviations

ARDS: Acute respiratory distress syndrome
ICU: Intensive care unit
SAPS: Simplified Acute Physiology Score
SD: Standard deviation
